# Preventive treatment effects on brain structures and functions in patients with chronic migraine: A multimodel magnetic resonance imaging study

**DOI:** 10.1002/kjm2.12903

**Published:** 2024-10-23

**Authors:** Tai‐Yuan Chen, Ching‐Chung Ko, Poh‐Shiow Yeh, Te‐Chang Wu, Yun‐Ju Shih, Chun‐Ming Yang, Ju‐Chi Lee, Ming‐Chung Chou, Kao‐Chang Lin

**Affiliations:** ^1^ Department of Radiology Chi Mei Medical Center Tainan Taiwan; ^2^ Graduate Institute of Medical Sciences Chang Jung Christian University Tainan Taiwan; ^3^ Department of Health and Nutrition Chia Nan University of Pharmacy and Science Tainan Taiwan; ^4^ Department of Neurology Chi Mei Medical Center Tainan Taiwan; ^5^ Department of Medical Sciences Industry Chang Jung Christian University Tainan Taiwan; ^6^ Department of Biomedical Imaging and Radiological Sciences National Yang‐Ming University Taipei Taiwan; ^7^ Institute of Healthcare Information Management National Chung Cheng University Chiayi Taiwan; ^8^ Department of Medical Imaging and Radiological Sciences Kaohsiung Medical University Kaohsiung Taiwan; ^9^ Department of Medical Research Kaohsiung Medical University Hospital Kaohsiung Taiwan; ^10^ Center for Big Data Research Kaohsiung Medical University Kaohsiung Taiwan; ^11^ Department of Neurology and Holistic Care Chi Mei Medical Center Tainan Taiwan

**Keywords:** functional connectivity, functional MRI, migraine, pain‐matrix network, structural connectivity

## Abstract

Patients with chronic migraine (CM) often exhibit structural and functional alterations in pain‐matrix regions, but it remains unclear how preventive treatment affects these changes. Therefore, this study aimed to investigate the structural and functional changes in pain‐matrix regions in CM patients after 6‐month treatment. A total of 24 patients with CM and 15 healthy controls were recruited for this study. Patients were divided into responder group (*N* = 9) and non‐responder group (*N* = 15). After completing the Migraine Disability Assessment (MIDAS) questionnaire, all patients underwent whole‐brain high‐resolution T1‐weighted images, diffusion‐weighted imaging, and resting‐state functional magnetic resonance imaging at baseline and 6‐month follow‐up. Whole brain gray matter volume and white matter diffusion indices were analyzed using voxel‐based analysis. Structural and functional connectivity analyses were performed to understand brain changes in patients after 6‐month preventive treatment. The responder group exhibited significantly higher MIDAS scores than the non‐responder group at baseline, but no significant difference between the two groups at follow‐up. No significant interval change was noted in gray matter volume, white matter diffusion indices, and structural connectivity in CM patients after 6‐month treatment. Nonetheless, the functional connectivity was significantly increased between occipital, temporal lobes and cerebellum, and was significantly decreased between parietal and temporal lobes after 6‐month preventive treatment. We concluded that resting‐state functional connectivity was suitable for investigating the preventive treatment effect on CM patients.

**Abbreviations**
AAL: automatic anatomical labeling
AD: axial diffusivity
AK: axial kurtosis
CAT12: Computational Analysis Tool version 12
DKE: Diffusion Kurtosis Estimator
DKI: diffusion kurtosis imaging
DTI: diffusion tensor imaging
FA: fractional anisotropy
FSL: FMRIB Software Library
ICHD‐3: International Classification of Headache Disorders, Third Edition
KFA: kurtosis fractional anisotropy
MD: mean diffusivity
MIDAS: Migraine Disability Assessment
MK: mean kurtosis
MNI: Montreal Neurological Institute
MRI: magnetic resonance imaging
PAG: periaqueductal gray
RD: radial diffusivity
RK: radial kurtosis
ROI: region‐of‐interest
rsfMRI: resting‐state functional MRI
S2: secondary somatosensory cortex
SPM12: Statistical Parametric Mapping version 12
TE: Echo Time
TR: Repetition Time
VBM: voxel‐based morphometry

## 1 BACKGROUND

Migraine is among the most prevalent neurological disorders worldwide, affecting more than 1 billion people.^1^ This disabling headache disorder can significantly impact patients' daily activities and quality of life.^2^ In the Global Burden of Disease study, migraine rose from seventh to second place in terms of years of disability between 2015 and 2016.^1^ A migraine diagnosis is based on the criteria described in the International Classification of Headache Disorders, Third Edition (ICHD‐3),^3^ and chronic migraine (CM) is diagnosed at least 3 months after onset and is characterized by headache days of 15 or more and migraine days of 8 or more in a month.^3^

Magnetic resonance imaging (MRI) is a non‐invasive technique with the potential to identify the morphological basis of neurological disease and investigate functional–structural relationships. Previous neuroimaging studies have demonstrated that patients with chronic migraine exhibit brain structural and/or functional alterations detected by diffusion tensor imaging (DTI),^4^ voxel‐based morphometry (VBM),^5^, ^6^, ^7^ and resting‐state functional MRI (rsfMRI) techniques.^8^, ^9^ These studies showed that CM patients exhibit decreased gray and white matter volume and higher white matter diffusion coefficient and functional connectivity, mostly in the pain matrix network.^10^ To investigate the treatment effect on CM patients, one previous study demonstrated that the decreased functional connectivity of default mode network was increased after acupuncture treatment in CM patients.^11^ Another event‐related fMRI study demonstrated that erenumab leads to decreased functional activation in the right thalamus after 2 weeks in patients with CM and episodic migraine.^12^

Although previous studies have shown that brain functions are altered in CM patients, it remains unclear how preventive treatment affects the brain structures and functions of the pain‐matrix network in CM patients. Our hypothesis is that preventive treatment may induce changes in brain structures and functions that are associated with headache symptoms. Therefore, the objective of this study was to investigate the impact of a 6‐month preventive treatment on the brain structures and functions of the pain‐matrix network in CM patients.

## 2 METHODS

### 2.1 Participants

This prospective study was approved by the local institutional review board of Chi Mei Medical Center (protocol number: 10905‐008). According to the ICHD‐3 criteria,^3^ we recruited 24 right‐handed CM patients (19 females and 5 males; mean age = 38.1 ± 7.9 years) and 15 age‐matched right‐handed healthy subjects (4 females and 11 males; mean age = 39.2 ± 11.4 years) for this study. To avoid potential confounding factors, participants with substance abuse, alcoholism, pregnancy, menstrual period, metal implants, or claustrophobia were excluded. After providing a signed informed consent, each patient underwent a clinical evaluation by skilled neurologists, which included the completion of the Migraine Disability Assessment (MIDAS) questionnaire^13^ and MRI acquisition at the time of enrollment and 6 months after treatment. In addition, the patients were further divided into responder group who had >30% decrease in MIDAS scores after treatment (8 females and 1 male) and non‐responder group (11 females and 4 males).^12^

### 2.2 MRI acquisition

All participants underwent MRI scans using a 3.0 T MR scanner (Discovery, MR750, GE Healthcare, USA), and the MRI scans were performed at least 48 h before and after a headache attack (inter‐ictal phase) for patients. First, whole‐brain T1‐weighted imaging data were acquired using a magnetization‐prepared rapid gradient‐echo sequence with the following parameters: TR = 5.57 ms, TE = 2.20 ms, TI = 450 ms, flip angle = 10°, field of view = 200 × 200 mm^2^, matrix size = 192 × 192, slice thickness = 1.0 mm, number of slices = 185, sagittal orientation, and number of excitations = 1. This imaging protocol was applied to both patient and control groups to evaluate the structural difference between the two groups. For CM patients, a series of whole‐brain rsfMRI data were further acquired using a gradient‐echo echo‐planar imaging pulse sequence with the following parameters: TR = 3000 ms, TE = 38 ms, matrix size = 64 × 64, number of slices = 48, slice thickness = 3 mm, field of view = 222 × 222 mm^2^ (in‐plane resolution = 3.5 × 3.5 mm^2^), acceleration factor = 2.0, and number of dynamics = 200. During the scan, subjects were asked to keep their eyes open, stay relax and not concentrate to a specific thought. Finally, whole‐brain diffusion data were acquired using a single‐shot, spin‐echo, echo‐planar diffusion‐weighted pulse sequence with the following parameters: TR = 5700 ms, TE = 90 ms, matrix size = 96 × 96, b = 1000 and 2000 s/mm^2^, number of slices = 48, number of diffusion directions = 30 for each high b‐value, number of b_0_ = 10, field of view = 240 × 240 mm^2^ (in‐plane resolution = 2.5 × 2.5 mm^2^), number of excitations = 1, acceleration factor = 2.0, and slice thickness = 3 mm. At the 6‐month follow‐up, each patient underwent a repeat MRI scan using the same imaging parameters with a range of 1 week before and after the treatment.

### 2.3 Voxel‐based morphometry

To detect gray matter changes in CM patients, the VBM analysis was carried out using the Computational Analysis Tool version 12 (CAT12, http://www.neuro.uni-jena.de/cat/). The VBM analysis involves several preprocessing steps, including field bias modulation, tissue segmentation, diffeomorphic anatomical registration through exponentiated lie algebra‐based spatial normalization,^14^ and spatial smoothing. Following spatial normalization and smoothing of the gray matter and white matter images, voxel‐wise analysis was performed to statistically compare the differences in gray matter and white matter between the two groups and in patients before and after the 6‐month treatment.

### 2.4 Voxel‐based DKI analysis

The DKI image processing involved several steps. First, FMRIB Software Library (FSL, https://fsl.fmrib.ox.ac.uk) linear image registration tool was used to perform affine registration and reduce eddy current distortions in the DWI images. Second, the Brain Extraction Tool was applied to remove non‐brain background signals from the imaging volume. Third, the Diffusion Kurtosis Estimator (DKE, https://www.nitrc.org/projects/dke/) toolbox was utilized to evaluate the DKI data and obtain various diffusion and kurtosis indices, including axial/radial/mean diffusivity (AD/RD/MD), fractional anisotropy (FA), axial/radial/mean kurtosis (AK/RK/MK), and kurtosis fractional anisotropy (KFA). Next, an international consortium of brain mapping–FA template images was used to spatially normalize individual FA maps and other diffusion maps using linear affine and non‐linear demon registrations. Finally, voxel‐based analysis was performed to compare the changes of diffusion indices in patients before and after treatment.

### 2.5 Structural connectivity network

The processing of the structural connectivity network involved several steps. First, affine registration was performed to co‐register the T1‐weighted images with the diffusion data. Second, 116 brain regions identified by an international consortium of brain mapping–T1 template images, known as the automatic anatomical labeling (AAL) regions, were spatially transformed to match the individual T1‐weighted images. Next, the displacement maps generated in the preceding stage were used to transform the 116 AAL regions. To determine structural connectivity, probabilistic tractography was performed using PROBTRACKX (FMRIB, Oxford, UK) with 5000 seeding points per voxel. Multiple fiber orientations of each voxel were estimated using a Bayesian estimator (BEDPOSTX, FMRIB, Oxford, UK) and were then used to trace neuronal fibers between two AAL regions. Finally, the structural connectivity between the two AAL regions was determined by dividing 5000 seeding points. The node strength, node degree, node‐local efficiency, and node‐clustering coefficient were measured from structural connectivity network.

### 2.6 Functional connectivity network

The rsfMRI data were processed using functional connectivity toolbox (CONN, https://web.conn-toolbox.org) running on a MATLAB platform (The MathWorks, Natick, Massachusetts, USA). The preprocessing steps included functional realignment and unwarping, slice‐timing correction, outlier identification, direct segmentation and normalization, and functional smoothing. To achieve functional realignment and unwarping, all scans were co‐registered and resampled to the first scan using b‐spline interpolation. To reduce temporal misalignment, the scan data were time‐shifted and resampled using sinc‐interpolation. During the outlier identification process, scans with displacements greater than 0.5 mm and standard deviations >3 were considered potential outliers. In direct segmentation and normalization, both T1‐weighted and rsfMRI data were spatially normalized to the standard Montreal Neurological Institute (MNI) space and then segmented into gray matter, white matter, and cerebrospinal fluid. Data denoising was conducted using linear regression and temporal band‐pass filtering with frequencies of 0.008 and 0.09 Hz. Finally, the correlation coefficient between two regions was used to evaluate functional connectivity using region‐of‐interest (ROI)‐to‐ROI approaches.

### 2.7 Statistical analysis

For clinical data, a Wilcoxon signed rank test was performed to assess the interval changes in the pain severity and MIDAS scores, and a Mann–Whitney *U*‐test was carried out to evaluate the difference between the two subgroups. For voxel‐based analysis, a two‐sample *t*‐test was performed to determine the difference in gray matter and white matter volume between patients and healthy controls at baseline and follow‐up scans. Besides, a paired *t* test was performed to determine the changes in gray matter volume and white matter diffusion indices in patients between the two scans. The results were considered significant if uncorrected *p* < 0.005 and cluster >100 voxels (cluster‐level corrected *p* < 0.05). For the structural and functional networks, a paired *t*‐test was performed to statistically compare the structural connectivity, node strength, node degree, node‐local efficiency, node‐clustering coefficient, and functional connectivity before and after treatment with age and sex as covariates. The comparisons were considered significant if corrected *p* < 0.05 with false discovery rate correction. Spearman correlation analysis was performed to reveal their correlations with MIDAS and pain severity, and the correlation was considered significant if *p* < 0.05.

## 3 RESULTS

### 3.1 Clinical and imaging features

After a 6‐month preventive treatment involving calcitonin gene‐related peptide (CGRP) antagonists, topiramate (anticonvulsants), and flunarizine (calcium channel blocker), the responder group exhibited significantly higher MIDAS scores than those of non‐responders at baseline, but no significant group difference after 6‐month treatment, as shown in Table 1. Moreover, during the study period, conventional MRI scans revealed no definitive space‐occupying lesion, no evident T2 hyperintensities in the white matter regions, and no apparent stenosis/spasm lesions, aneurysms, arteriovenous malformations, or arteriovenous fistulas in the patients' circle of Willis.

**TABLE 1:** The demographic characteristics of enrolled patients with chronic migraine at the time of enrollment (first) and 6‐month follow‐up (second).

	Age (years)	Disease duration (years)	Pain severity (0–10)		MIDAS score	
			First	Second	First	Second
All (M:F = 5:19)	38.1 ± 7.9	18.3 ± 11.0	6.0 ± 2.2	5.6 ± 1.8	32.6 ± 48.3	24.2 ± 35.5
Responder (M:F = 1:8)	39.1 ± 9.2	22.3 ± 12.2	4.6 ± 1.6	4.6 ± 1.6	57.3 ± 42.1^a^, ^b^	16.3 ± 17.5
Non‐responder (M:F = 4:11)	37.2 ± 7.9	16.1 ± 10.6	6.4 ± 2.1	5.9 ± 1.7	7.9 ± 17.0^b^	23.0 ± 37.7

Abbreviation: MIDAS, Migraine Disability Assessment.

^a^ Indicate significant difference between two groups.

^b^ Indicate significant difference between baseline and follow‐up (*p* < 0.05).

### 3.2 Voxel‐based morphometry

**FIGURE 1:**
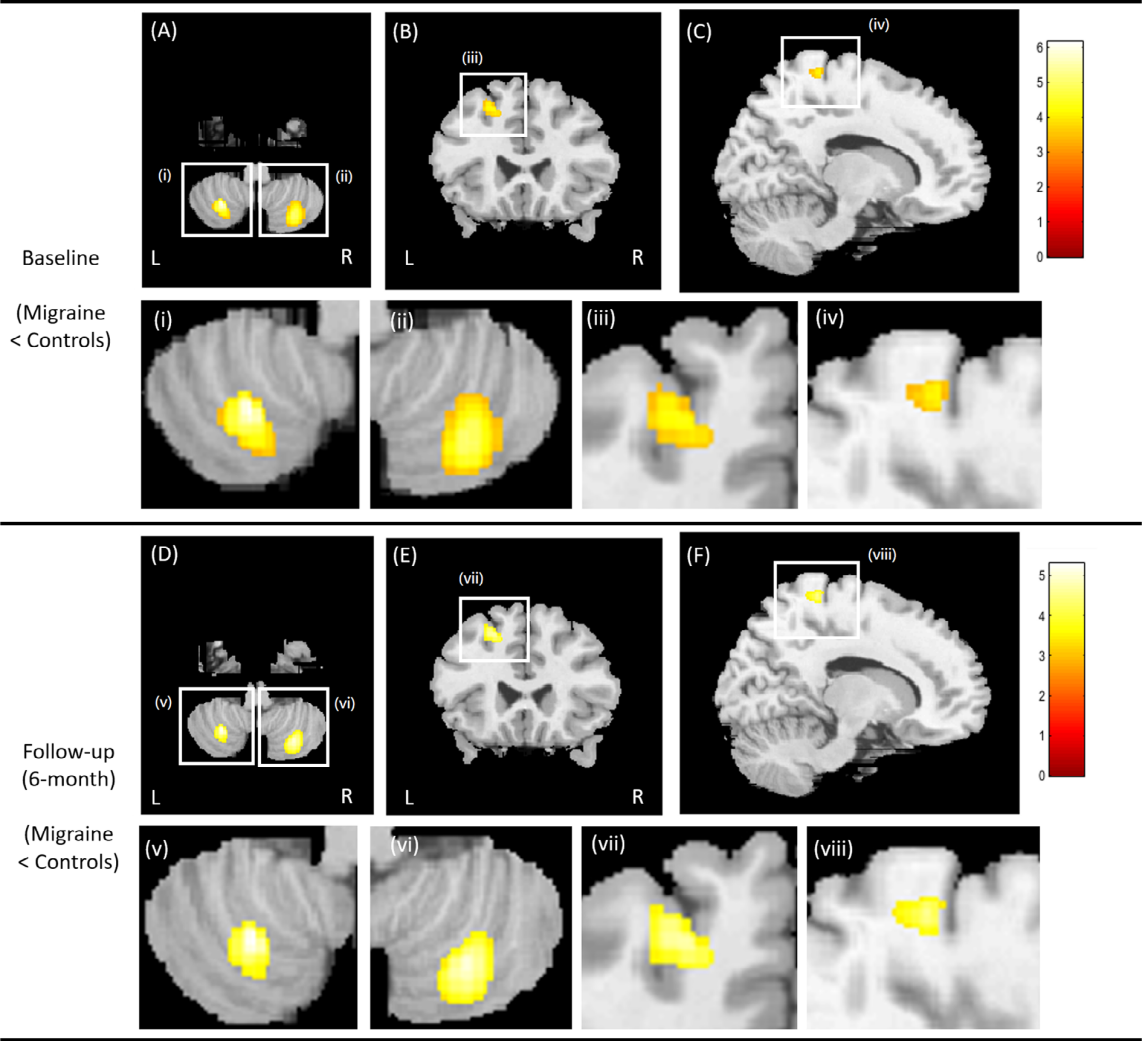
Voxel‐based comparison of gray matter volume between the migraines and healthy controls at baseline (A–C) and follow‐up scans (D–F). The significant regions were enlarged for better visualization at baseline (i–iv) and follow‐up (v–viii). In VBM analysis, the gray matter volumes were estimated on a voxel‐by‐voxel basis, and the volumes were compared between the two groups for the whole brain areas. After statistical analysis, those regions (or imaging voxels) with statistical significance were displayed in yellow‐to‐red colors and superimposed on MR images. The red indicates lower T score (lower significance) and yellow indicates higher *T* score (higher significance). The *T* value is indicated by the color bar on the right.

At baseline, the comparisons indicated a significant decrease in gray matter volume in several regions in the patients compared to healthy controls. These regions included the bilateral cerebellum, left middle frontal gyrus, left cuneus, right supramarginal gyrus, right postcentral gyrus, and right hippocampus. In the follow‐up, the migraines continued to exhibit a significant decrease in gray matter volume in the bilateral cerebellum, bilateral temporal lobe, left middle frontal gyrus, right supramarginal gyrus, right postcentral gyrus, and right lingual gyrus, as depicted in Figure 1. However, no significant difference of white matter volume was noted between CM patients and healthy controls.

**FIGURE 2:**
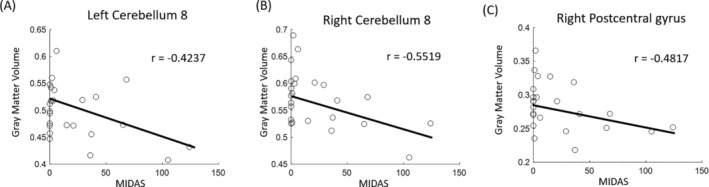
Significant correlations between gray matter volume and MIDAS scores in the left cerebellum 8 (A), right cerebellum 8 (B), and right postcentral gyrus (C) in patients after 6‐month treatment.

In significant regions, correlation analysis revealed significant negative correlations between gray matter volume and MIDAS scores in the bilateral cerebellum (*r* = −0.4237 and −0.5519 for the left and right sides with *p* = 0.044 and 0.006, respectively), as well as in the right postcentral gyrus (*r* = −0.4817 and *p* = 0.02), as shown in Figure 2. However, no significant difference of gray matter volume was noted in the patients between the baseline and follow‐up.

### 3.3 Voxel‐based DKI analysis and structural connectivity

The voxel‐based DKI analysis did not reveal any significant changes in diffusion indices in CM patients after a 6‐month treatment. Additionally, the graph‐theory analysis of the structural connectivity network did not detect any significant changes in structural connectivity, node strength, node degree, node clustering coefficient, or node local efficiency in the migraines after the 6‐month preventive treatment.

### 3.4 Functional connectivity network

**FIGURE 3:**
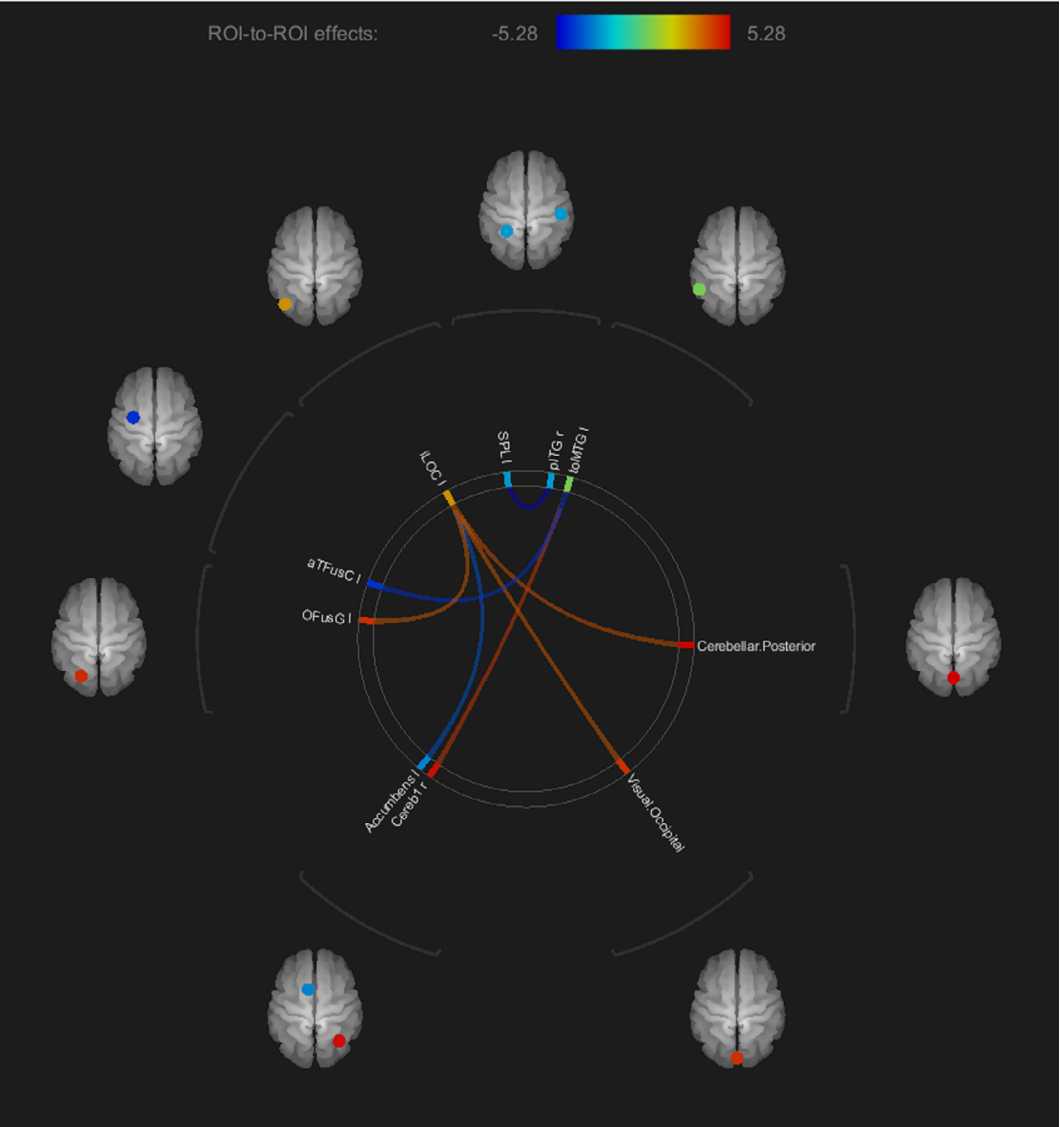
The significant increases (red connections) and decreases (blue connections) of functional connectivity between the brain regions in the migraines after 6‐month treatment.

The results revealed significant increases in functional connectivity in CM patients after a 6‐month follow‐up. Specifically, there was a significant increase in connectivity between the following regions: the left inferior division of the lateral occipital cortex (iLOC), posterior cerebellum, left visual occipital cortex, and left occipital fusiform gyrus (OFusG); as well as between the left temporooccipital part of the middle temporal gyrus (toMTG) and the right cerebellum crus 1. These findings are illustrated in Figure 3. Furthermore, significant increases in connectivity were observed between the left toMTG and the left anterior division of the temporal fusiform cortex (aTFusC), between the left iLOC and the accumbens, and between the left superior parietal lobule (SPL) and the right posterior division of the inferior temporal gyrus (pITG) among migraines after the 6‐month follow‐up.

**FIGURE 4:**
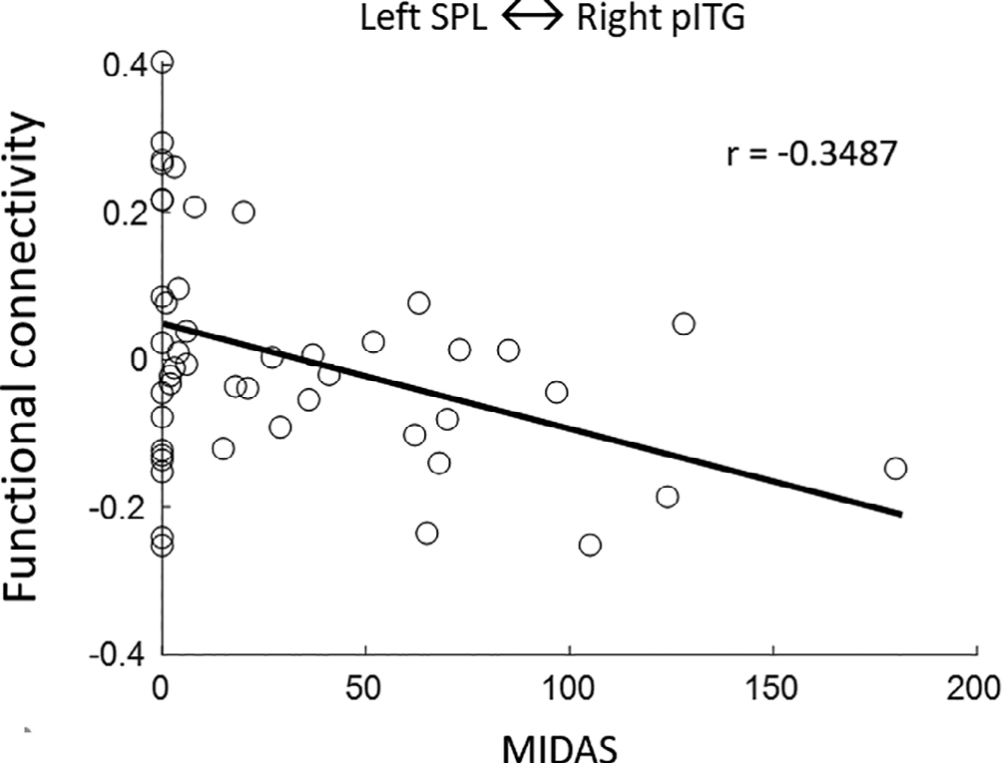
The relationships between functional connectivity and MIDAS scores in the connection between left superior parietal lobule (SPL) and right posterior division of the inferior temporal gyrus (pITG).

In significant regions, a significant negative correlation was observed between the MIDAS score and the connection between the left SPL and right pITG (*r* = −0.3478 and *p* = 0.018), as shown in Figure 4.

**FIGURE 5:**
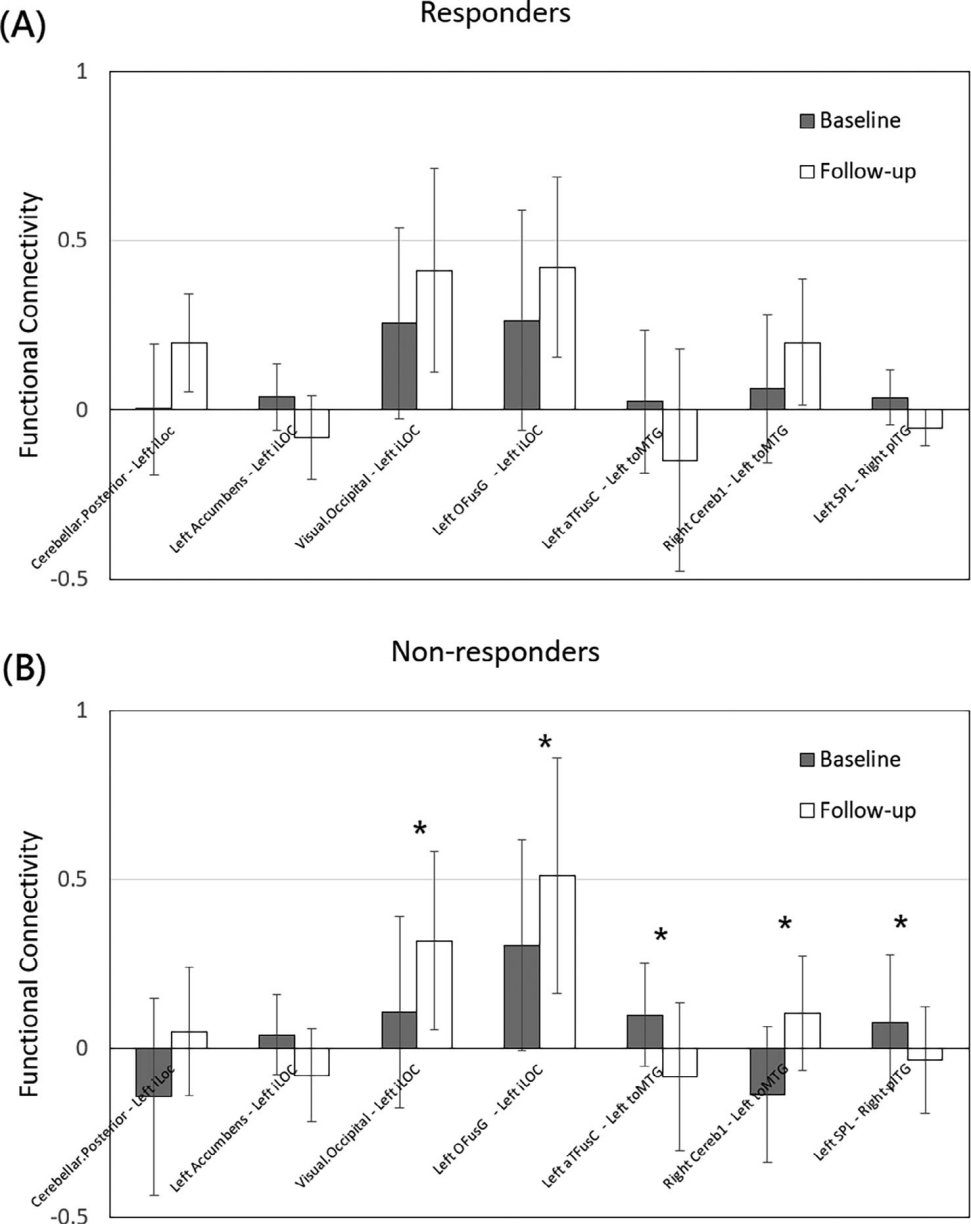
The interval changes of functional connectivity in the responders (A) and non‐responders (B). Asterisks (*) indicate significant difference (*p* < 0.05).

Moreover, the analysis revealed that the responders exhibited significantly higher functional connectivity than those of non‐responders in the connection between left iLOC and posterior cerebellum (0.2 ± 0.14 vs. 0.05 ± 0.19, *p* = 0.037) at 6 months after treatment, and that the non‐responders exhibited significant interval changes of functional connectivity in five different connections, but no significant interval change was noted in the responders, as shown in Figure 5.

## 4 DISCUSSION

To the best of our knowledge, this is the first study to investigate the interval changes of brain structural and functional connectivity networks in patients with chronic migraine before and after a 6‐month preventive treatment. Previous studies have shown that CM patients had lower gray matter volume in multiple pain‐related brain regions compared to healthy controls.^15^, ^16^ In line with previous studies, we found that gray matter volume was significantly reduced in the bilateral cerebellum, left middle frontal gyrus, and right postcentral gyrus of migraine at both baseline and the 6‐month follow‐up. Within these significant regions, gray matter volumes had negative correlations with MIDAS scores in the bilateral cerebellum and right postcentral gyrus. However, no significant difference was noted in white matter volume between the two groups at both baseline and follow‐up. These findings suggest that headache symptoms were associated with gray matter reduction in the cerebellum and postcentral gyrus in migraines after the 6‐month preventive treatment.

In white matter diffusion indices and structural connectivity, the present study did not reveal any significant interval changes of white matter diffusion indices and structural connectivity network in CM patients after 6‐month treatment, nor the difference between the responder and non‐responder groups. These findings suggest that the 6‐month preventive treatment may not be long enough to cause significant changes of white matter diffusion indices and structural connectivity in CM patients. However, in the functional connectivity, previous studies have shown that CM patients exhibit decreased functional connectivity in the dorsolateral and medial prefrontal cortices, precuneus, and amygdala. Conversely, they have demonstrated increasing functional connectivity in the orbital frontal gyrus, temporal pole, hippocampal complex, cerebellum, and between the hypothalamus and regions belonging to the default mode network and dorsal visual network.^17^, ^18^, ^19^ Additionally, other studies have indicated that CM patients have decreased functional connectivity in the default mode network, salience network, and central executive network.^9^, ^20^ Although previous studies performed fMRI to investigate the effect of acupuncture and erenumab treatments in CM patients,^11^, ^12^ no previous study has utilized rsfMRI to investigate the interval changes of functional connectivity in pain‐matrix network after a 6‐month preventive treatment in CM patients. The present study revealed that functional connectivity was significantly increased between the occipital, parietal, and cerebellum regions in CM patients, but significantly decreased between the temporal and parietal lobes after 6‐month treatment. Furthermore, within these significant connections, a negative correlation was found between the MIDAS score and the connection of the left SPL and the right pITG in migraines, suggesting that the interval changes of headache symptoms are associated with functional connectivity between pain‐related regions.

Further clinical evidence is necessary to fully comprehend the pathophysiological mechanism of treatment effects on CM patients. The present study demonstrated that the responder group exhibited significantly higher MIDAS scores than those of non‐responder group at baseline, but no significant difference was noted between two groups at follow‐up. Our imaging results showed that the responders exhibited significantly higher functional connectivity in the connection between occipital lobe and cerebellum than those of non‐responders at 6 months after treatment. These findings suggest that functional connectivity between occipital and cerebellum might play a pivotal role in treatment effect on CM patients. Moreover, the comparisons further revealed that non‐responders exhibited significant changes in functional connectivity across five connections (Figure 5B), accompanied by a significant increase in MIDAS scores after the 6‐month treatment. In contrast, responders showed no significant changes in functional connectivity, along with a significant decrease in MIDAS scores after the same treatment period. These findings suggest that the significant changes in functional connectivity within these connections among non‐responders may be attributed to a deterioration in headache symptoms indicated by the increased MIDAS scores.

This study has several limitations that warrant discussion. First, the sample size was small in this preliminary study, highlighting the need for future studies to include a larger number of subjects. Second, the sex was not well matched between CM patients and healthy controls, so the results may be affected by the sex difference. Third, the enrollment of healthy subjects was limited to understanding the alterations of gray matter and white matter volumes in CM patients. Fourth, the headache frequency was not analyzed because patients did not use headache diary to record it on a daily basis during the study period. Lastly, imaging during spontaneous migraine attacks proved challenging due to the episodic nature and discomfort associated with such attacks. Consequently, the present study conducted MRI acquisitions on patients between attacks, specifically during the interictal phase (defined as a period of more than 48 h between headache phases).

## 5 CONCLUSIONS

In the present study, we compared gray matter volume, white matter diffusion indices, and structural and functional connectivities in CM patients before and after a 6‐month preventive treatment. Our results revealed no structural changes after the 6‐month treatment, but significant changes in functional connectivity were observed in pain‐matrix regions, which were correlated with headache symptoms in migraines. Consequently, we concluded that resting‐state functional connectivity was suitable for investigating the preventive treatment effect on CM patients.

## CONFLICT OF INTEREST STATEMENT

The authors declare that they have no competing interests.

## Data Availability

All patients' data are not available due to privacy issue and institutional regulations.
