# Correction to “Preventive Treatment Effects on Brain Structures and Functions in Patients With Chronic Migraine: A Multimodel Magnetic Resonance Imaging Study”

**DOI:** 10.1002/kjm2.70016

**Published:** 2025-04-08

TY Chen, CC Ko, PS Yeh, TC Wu, YJ Shih, CM Yang, JC Lee, MC Chou, and KC Lin, “Preventive Treatment Effects on Brain Structures and Functions in Patients With Chronic Migraine: A Multimodel Magnetic Resonance Imaging Study,” *The Kaohsiung Journal of Medical Sciences* 40, no. 12 (2024): 1077–1085, https://doi.org/10.1002/kjm2.12903.

On page 1080, in Section 3.4 Functional connectivity network, the original text “Furthermore, significant increases in…” contains an error and should be corrected to “Furthermore, significant decreases in…” The word *increases* should be replaced with *decreases*.

The authors confirmed that all results and conclusions of this article remain unchanged.

We apologize for this error.

